# Heat stress impact on rice reproductive processes: challenges and new approaches

**DOI:** 10.3389/fgeed.2026.1808390

**Published:** 2026-08-24

**Authors:** Miguel Moreira, Ana Rita Queirós, Ana Ventura, Diana Moreira, Sílvia Coimbra, Ana Marta Pereira

**Affiliations:** LAQV/REQUIMTE, Biology Department, Faculty of Sciences, University of Porto, Porto, Portugal

**Keywords:** breeding strategies, climate change, gene editing, *Oryza sativa* (L.), plant reproduction, pollen-pistil interactions

## Abstract

Heat stress represents one of the most severe abiotic constraints to rice (*Oryza sativa* L.) productivity and is expected to intensify under ongoing climate change, particularly affecting the reproductive phase and leading to substantial yield and grain quality losses. This review synthesizes current knowledge on the impacts of heat stress on rice reproduction, with a focus on both male and female reproductive structures and their interactions. Evidence from anatomical, physiological, transcriptomic, and metabolomic studies to describe how elevated temperatures disrupt key reproductive processes, including microsporogenesis, anther dehiscence, pollen viability, pollen-pistil interactions, fertilisation, and embryo sac development were integrated in this review. It further discusses the genotype-dependent differences in reproductive thermotolerance; and key genes, metabolites, and pathways associated with heat stress perception, signalling, and tolerance are highlighted. Finally, it is briefly discussed how recent advances in breeding strategies, functional genomics and genome-editing technologies, particularly CRISPR-based approaches, are providing new opportunities to enhance reproductive resilience to heat stress and how it is essential to close the existing molecular knowledge gaps in the development of heat-tolerant rice varieties capable of sustaining productivity in a warming climate.

## Introduction

Over the past few centuries, the global population has grown rapidly and is projected to reach 8.5 billion by 2030. This demographic expansion is expected to intensify pressure on agricultural land and food production systems, which are already under strain due to climate change (CC) (Arora, 2019; Shaheen et al., 2023). Climate refers to the long-term average of weather conditions in a specific region, whereas CC, driven by both natural and anthropogenic activities, denotes significant shifts in these patterns. The interaction between humans and nature has always been dynamic; in early societies, survival depended directly on natural ecosystems (Muluneh, 2021; Abbass et al., 2022). However, with the emergence of agriculture, approximately 12,000 years ago, it enabled the establishment of permanent settlements, which substantially increased the human impact on the environment (Carey, 2023). Nowadays, agriculture remains the basis of global food security and economic stability but is also one of the sectors most vulnerable to CC (Mendelsohn, 2009). CC is recognised as one of the defining challenges of the 21st century, with profound implications in food systems, human health, and sustainable development. Projections from the Intergovernmental Panel on Climate Change (IPCC) indicate that global mean temperatures could rise by 1.5 °C–2 °C above pre-industrial levels by mid-century, intensifying extreme weather events, altering precipitation regimes, and accelerating sea-level rise (IPCC, 2018; IPCC, 2021; Calvin et al., 2023). Forecasts for global climate indicate that temperatures are likely to remain at or close to record highs over the next 5 years, heightening climate-related risks and their effects on societies, economies, and sustainable development (WMO, 2025). All of these are expected to coincide with a 50%–85% increase in global food demand in the coming decades (Raza et al., 2019; FAO et al., 2022). However, agricultural production is unlikely to match this demand, as climate-related stresses (abiotic stresses) are projected to reduce crop yields by up to 30%, threatening the sustainability of food systems worldwide (González-Schain et al., 2016; Massel et al., 2021). Among these stresses, heat stress (HS) is particularly detrimental to plant growth and development (Hatfield and Prueger, 2015; Gillison, 2019). Its impact varies across developmental stages, and this has been thoroughly reviewed (Li et al., 2022; Ren et al., 2023; Xing et al., 2024; Ma et al., 2025), but the reproductive phase (reproduction and grain development/filling) is especially sensitive, when even short periods of stress can severely compromise fertilization, grain set and final yield (Benitez-Alfonso et al., 2023). Rice (*Oryza sativa* L.), a fundamental cereal crop that sustains nearly half of the global population, is cultivated in more than 100 countries with an annual production exceeding 780 million tons (Fahad et al., 2019). Beyond its agricultural importance, rice has also become a model specie for monocotyledon research, due to several biological and practical attributes, such as, small and sequenced genome, self-fertilisation, a short annual life cycle despite its capacity to survive longer under favourable conditions, ease of cultivation, efficient *Agrobacterium*-mediated transformation, and the availability of diverse varieties and mutants (Sasaki and Burr, 2000). Despite that, extreme weather events pose a major challenge to rice productivity. Currently, 16% of rice-growing areas experience critical HS during the reproductive stage, a figure projected to rise to 27% by 2050 (Gourdji et al., 2013). For each 1 °C increase above the threshold is expected to reduce yields by about 3.2% (Fu et al., 2016; Arshad et al., 2017). Therefore, this review focuses specifically on the reproductive phase, where HS has the most immediate consequences for yield formation and food security. We summarize current knowledge on how HS affects rice reproduction, with particular emphasis on the molecular mechanisms and genetic determinants underlying reproductive thermotolerance. We further discuss current challenges and emerging strategies, including genomics assisted and genome editing approaches, to support the development of heat-resilient rice varieties.

## Rice reproduction

The physiological and molecular processes involved in flowering plants’ sexual reproduction still hold many secrets, especially when challenged by the growing threat of environmental stresses such as high temperatures (Pereira and Coimbra, 2019; Silvestro et al., 2021). Rice reproduces sexually, and as in many other species, its economic value lies in the outcome of its reproductive phase, that is, the seed/grain (Hedhly, 2011). The main factors determining rice yield are the number of grains per panicle, the rate of seed setting within the panicle, the number of panicles per plant, and the individual grain weight (Zhao et al., 2025).

**FIGURE 1:**
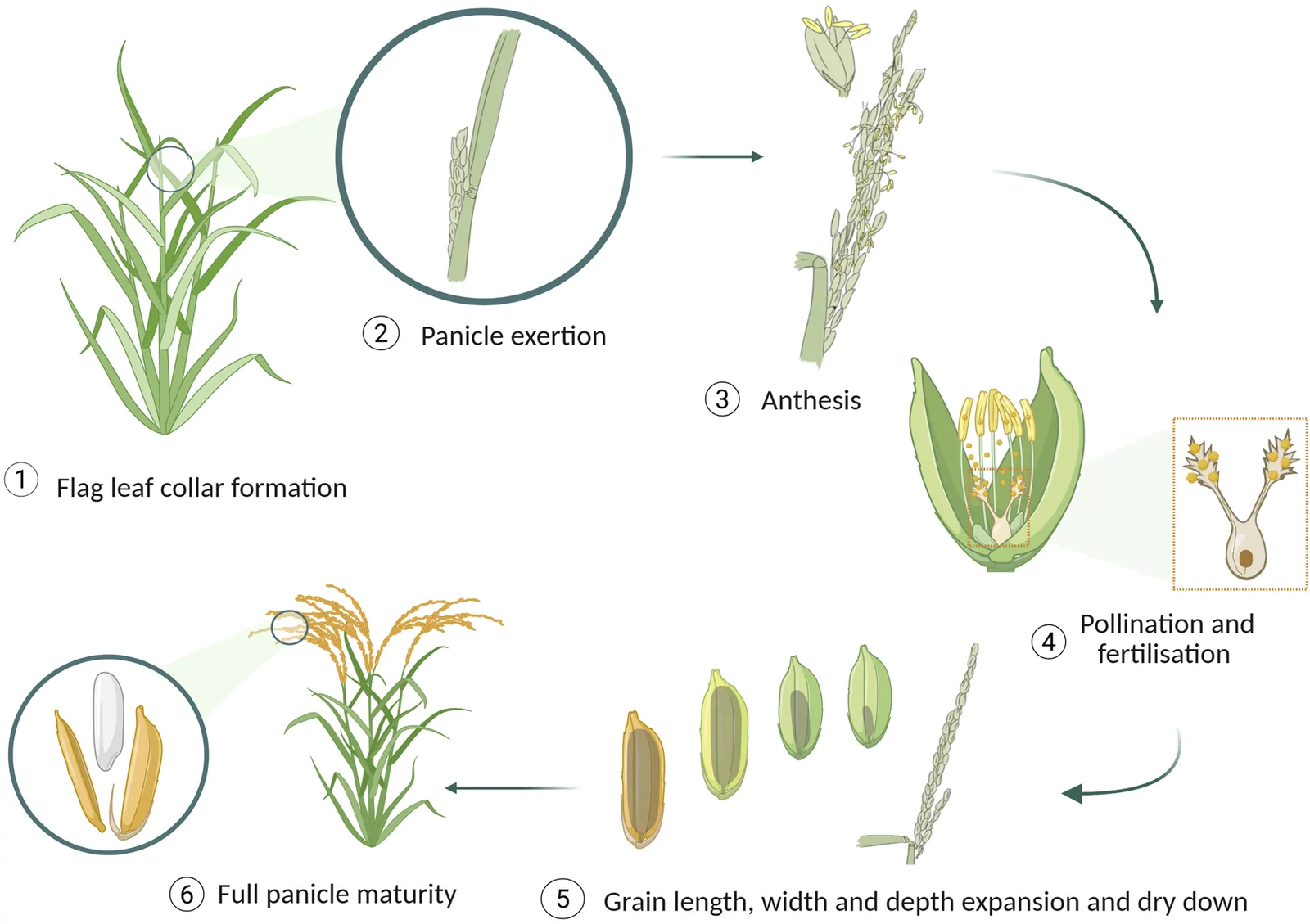
Schematic representation of important steps in the reproductive phase of rice plants. 1) Flag leaf collar formation–the final leaf (flag leaf) emerges, with its collar forming around the developing panicle, enclosed within the stem; 2) Panicle exertion–the panicle tip emerges from the flag leaf sheath; 3) Anthesis–flowers open and the anthers protrude from the spikelet, due to stem elongation, releasing the pollen; 4) Pollination and fertilisation–pollen is deposited in the stigma, followed by pollen tube growth and transportation of the sperm cells into the ovary to accomplish double fertilization and initiate seed/grain development; 5) Grain length, width and depth expansion and dry down–upon fertilization, the grain fills with starch (increasing length, width, depth) and gradually dries until it reaches maturity, transitioning through milk and dough stages. 6) Full panicle maturity–at this stage, 90%–100% of the grains have completed dry matter accumulation. The endosperm is fully hardened, the grains have reached their final morphology, the glumes have yellowed, and grain moisture content has decreased to around 20%–25%. Created with BioRender. Pereira, A. M. (2026) https://BioRender.com/m7789ob.

In rice, the reproductive phase (Figure 1) begins with the transition of the shoot apical meristem from the vegetative to the reproductive development, leading to panicle initiation. A panicle is a conical raceme in which spikelets are borne on both primary and secondary branches extending from the rachis (Bell, 1991). A typical panicle contains more than ten primary branches and approximately 150 spikelets. The development of the panicle proceeds through several well-defined stages that include panicle initiation, panicle differentiation, flag leaf collar formation, panicle exertion, anthesis, grain expansion in length, width and depth, grain dry down, single grain maturity, and full panicle maturity (Ikeda et al., 2004; Caselli et al., 2020). Each spikelet contains a single fertile floret flanked by a pair of sterile lemmas. The floret is composed of the lemma and palea, bract-like organs, two lodicules that function as modified petals, six stamens, and a single carpel forming the pistil (Guo et al., 2015). The carpel encloses the ovule, which originates from integument primordia and develops an embryo sac containing one egg cell, two synergids, a diploid central cell, and three antipodal cells (Lopez-Dee et al., 1999; Ikeda et al., 2004). Male (pollen grains) and female gametophytes (embryo sac) form through meiosis of the respective spore mother cells, followed by mitotic divisions and gametophyte maturation.

Pollination and fertilisation in rice are highly synchronised processes. During anthesis, rice flowers typically open around midday, remaining open for approximately 15 minutes under summer conditions, during which the anthers dehisce and release pollen onto the receptive stigma (Satoh and Omura, 1979), leading to fertilisation within a few hours. The rice pollen is highly sensitive, retaining high water content at the time of release and losing viability within minutes of exposure to air. This recalcitrant nature requires rapid pollen capture and hydration for successful fertilisation (Nepi et al., 2001; Pacini et al., 2006; Pacini and Dolferus, 2019; Moon and Jung, 2020). Once on the stigma, pollen grains (PGs) germinate and extend the pollen tubes (PTs) through the style transmitting tract, until reaching the ovule, to deliver two sperm cells to the embryo sac, deeply embedded in the maternal tissues. One sperm fuses with the egg cell to form the zygote, while the other fuses with the central cell to produce the primary endosperm nucleus. This double fertilisation, characteristic of angiosperms, ensures the coordinated development of both embryo and endosperm, the latter being the main storage tissue of the rice grain.

Following fertilisation, the zygote undergoes successive cell divisions to form the embryo, while the endosperm proliferates and accumulates starch. At the same time, maternal tissues, including the lemma, palea, and seed coat develop the caryopsis, providing structural protection. Grain filling is marked by the expansion of the caryopsis and the deposition of starch within endosperm cells, accompanied by the differentiation of the aleurone layer (Counce and Moldenhauer, 2019). As the seed matures, chlorophyll in surrounding maternal tissues is degraded, the seed undergoes desiccation, and it eventually enters a quiescent state. Germination begins when external conditions become favourable, particularly with adequate water supply and optimal temperatures between 15 °C and 35 °C, with 25 °C as the optimum (Yang et al., 2019).

Rice reproduction, from floral initiation through seed setting, encompasses a finely regulated sequence of developmental events. Each stage, from panicle formation to pollination, fertilisation, and grain filling, is essential for determining yield and is highly sensitive to environmental conditions. These developmental transitions that terminate one growth phase and initiate another are crucial events in complex multicellular organisms. In plants, these transitions enable adaptive responses to abiotic or biotic environmental cues (Huijser and Schmid, 2011).

## Heat stress impacts reproduction in rice

Recent declines in rice planting have been noted in Europe due to water scarcity and climate shifts. For example, in Portugal main rice-growing regions (Tejo, Sado and Mondego estuaries) have seen a reduction in production in recent years, which can only be compensated by an increase in cultivation area and water availability (COTArroz, 2023). Based on all conducted research and recorded production losses, it was calculated that for every degree Celsius increase, rice grain yield fell by 10% (Prasad et al., 2006). By 2030, rice production will need to rise by 40% to meet the rising demand of a growing global population. So, enhancing rice productivity is imperative to align with the escalating demands driven by demographic factors (Jagadish et al., 2015; Shaheen et al., 2023).

**FIGURE 2:**
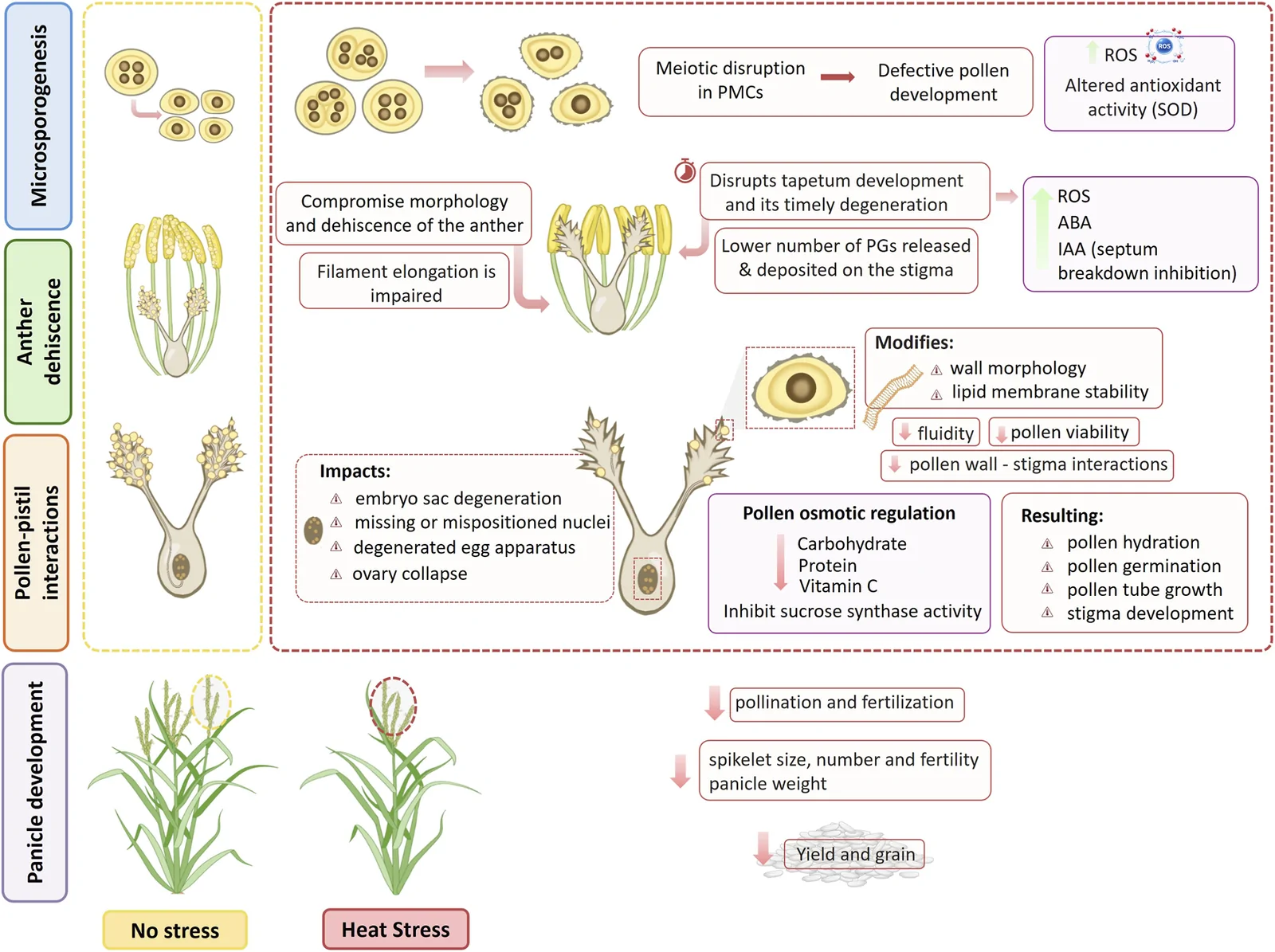
Effects of heat stress (HS) on rice reproductive development and pollen–pistil interactions. Schematic overview summarising the different impacts of HS on rice reproduction, including cellular, tissue, organ, and whole-plant responses. High temperatures disrupt key processes during male gametophyte development, including meiotic progression in pollen mother cells (PMCs), tapetum development and degeneration, filament elongation, and anther dehiscence, resulting in reduced pollen viability and pollen release. At the cellular level, HS induces oxidative stress, hormonal imbalances, metabolic and osmotic dysregulation, and membrane and cell wall instability, which collectively impair pollen function and pollen–stigma interactions. Female reproductive tissues are also affected by HS, leading to embryo sac degeneration, mispositioned nuclei, degeneration of the egg apparatus, and ovary collapse, severely affecting pollen–pistil interactions: HS impairs pollen hydration, germination, pollen tube growth, and stigma development. The disruption of both male and female reproductive processes ultimately leads to reduced pollination and fertilisation efficiency, leading to decreased spikelet number, fertility, panicle weight, and grain yield. While male reproductive responses to HS have been extensively studied, this figure highlights the overlooked contribution of female reproductive tissues to reproductive failure under HS.

For most crop species, the success of reproductive development determines yield, and the major environmental factor that affects this is temperature (Hatfield and Prueger, 2015). It was observed across different species that high temperatures disrupt the development of anther and pistil structures, compromise pollen and ovules integrity, limit gamete development, suppress pollen formation, and restrict PT growth and pollen-pistil interactions, which affects processes, such as pollination, fertilisation, endosperm formation and embryo development (Figure 2) (Snider et al., 2011; Giorno et al., 2013; Begcy et al., 2024).

Within *O. sativa*, two subspecies are distinguished, *indica* and *japonica* (Johns and Mao, 2007), which display notable differences in their responses to HS. While heat-tolerant genotypes have been identified in both subspecies, *indica* varieties are generally regarded as more tolerant to high temperatures than *japonica*, having different morphological and physiological characteristics (Matsui, 2000; Wang et al., 2016; Perkins et al., 2025). The exposure to HS conditions (35 °C) in different rice genotypes leads to reduced anther dehiscence, a lower number of PGs deposited on the stigma, and a decrease in pollen germination (Das et al., 2014; Cai et al., 2020). The impact of HS varies across developmental stages. Early vegetative and reproductive stages are comparatively less affected, partly due to buffering effects provided by floodwater, whereas gametogenesis and flowering represent the most heat-sensitive phases (Prasad et al., 2006; Jagadish et al., 2007; 2008; 2015). Rice panicle development is also impacted by HS, which results in fewer and smaller spikelets, lowering the panicle’s weight. However, little is known about how it affects other developmental stages, such as the growth of early floral meristems, spikelet differentiation, pollen-pistil interactions, and grain filling.

In general, the reproductive consequences of HS have been more extensively studied in male tissues than in female tissues. When compared to the ovule, pollen viability and anther development are more susceptible to the warmth inside the floret (Shrestha et al., 2022). Although male gametophytes are considered more sensitive than female gametophytes, pistil development under HS remains an important aspect that warrants further attention. HS can compromise morphology and dehiscence of the anther, reduce pollen viability and deposition on stigmas, impair pollen hydration, germination, and tube growth, and even alter stigma development. Collectively, these impairments hinder pollination and fertilization, ultimately leading to reduced spikelet fertility (Jagadish et al., 2010; Das et al., 2014; Tazib et al., 2015; Fu et al., 2016; Xu et al., 2020; Shrestha et al., 2022; Guan et al., 2025).

## Impact of heat stress on male reproductive development and anthesis

Among the reproductive organs, the male structures are consistently reported as the most sensitive to HS. In rice, the stamen filament elongation, which enables anther emergence from the glume and normally occurs in coordination with lodicule swelling, is a key process for successful fertilization (Heslop-Harrison and Heslop-Harrison, 1996). However, under HS this elongation is impaired, resulting in delayed growth (Katano et al., 2020; Yao et al., 2024). One of the subsequent processes, microsporogenesis, is highly affected by HS, with studies indicating that specially the tetrad stage, exhibits significant sensitivity to HS in cereal crops, including rice (Liu et al., 2023). HS disrupts the meiotic division of pollen mother cells (PMCs) during sporogenesis, thereby impairing PG development (Visakh et al., 2024). Supporting this evidence, rice plants exposed to high temperatures (41 °C during the day and 30 °C at night) for 10 days at the PMC meiosis stage showed a pronounced 78.8% reduction in pollen viability and a 48.5% decrease in seed set compared with plants grown under control conditions (30 °C during the day and 24 °C at night) (Hossain et al., 2024). This sensitivity has been linked to significant changes in cellular homeostasis, including increased levels of reactive oxygen species (ROS) and altered antioxidant activity, such as superoxide dismutase (SOD), within the anthers under HS (Qian et al., 2025). Beyond the disruption of microsporogenesis, HS also compromises profound structural abnormalities in both anthers and pollen. Notably, even a single day of HS can alter anther structure and pollen wall morphology, further compromising pollen viability (Liu et al., 2023). At the molecular level, several regulators link metabolic homeostasis and stress response during pollen meiosis. *GROWTH-REGULATING FACTOR 4* (*OsGRF4*), a miR396-regulated gene acting during pollen meiosis, integrates nitrogen and carbon metabolism. The *OsGRF4* ^*AA*^ variant escapes miR396-mediated degradation, altering its expression and splicing. Under HS, this allele is associated with stronger repression of genes involved in carbohydrate metabolism, photosynthesis, lipid metabolism, and key regulators of anther development, including male-sterility genes. Overall, *OsGRF4* variation disrupts transcriptional and post-transcriptional regulation during pollen meiosis, impairing nutrient balance in anthers and reducing heat tolerance (Mo et al., 2023). Similarly, a *GLUTAMYL-TRNA SYNTHETASE 1–2 (ERS1-2*) gene is required for normal meiotic development. *esr1-2* mutant has been reported to cause severe delays in pollen development and meiosis under HS conditions, resulting in male infertility (Liu et al., 2024). Other genes, such as *HEAT SHOCK PROTEIN60-3B (OsHSP60-3B)* and its interactor *FLOURY ENDOSPERM6* (*FLO6*) were identified through the characterisation of the heat-sensitive male-sterile rice mutant *oshsp60-3b*, in which loss of OsHSP60-3B function causes temperature-dependent male sterility that becomes progressively more severe with increasing temperature. In contrast, *OsHSP60-3B* overexpression lines exhibit enhanced pollen thermotolerance, supporting its role as a positive regulator of heat tolerance in rice. The authors showed that *OsHSP60-3B* is rapidly induced by heat shock and localises to plastids, where it interacts with *FLO6*, a key component involved in starch granule formation in rice pollen. This interaction appears to be required for the stabilisation of FLO6 under high-temperature conditions. Through this mechanism, *OsHSP60-3B* contributes to the maintenance of pollen starch granule biogenesis and to the attenuation of ROS accumulation in anthers, thereby preventing cell death and pollen abortion and ensuring normal male gametophyte development under HS (Lin S. et al., 2023). *REDUCED HEAT STRESS TOLERANCE 1 (OsRHS*), was identified as negatively regulating heat tolerance in rice, at least partly by impairing pollen development during the flowering stage (Mao et al., 2025). Another study demonstrated that *ONAC023*-mediated heat tolerance is also linked to the regulation of pollen fertility during the reproductive stage in rice (Chang et al., 2024) reinforcing the idea that HS strongly affects pollen development at flowering.

In angiosperms, anther dehiscence is a critical reproductive step that allows pollen to be released for pollination. The timing of pollen release is influenced by temperature, and most plants will break at a particular temperature. Pollen release may be impacted, and pollination success could be compromised if dehiscence happens earlier or later than usual under high-temperature stress. In rice, anther dehiscence occurs at both its apical and basal ends, with the basal opening playing a particularly important role under HS (Matsui et al., 2005; Jagadish et al., 2010). In terms of structure anatomy, during anthesis, the basal pores open as the anthers assume an erect position, and the length of these pores varies among rice cultivars. This variation is important because pollen release onto the stigma during anther dehiscence is closely related to basal pore size, with longer basal pores generally allowing a greater number of PGs to be discharged onto the stigma, while in cultivars with smaller basal pore the PGs remained trapped inside the anthers until get disperse by wind when the floret opens. Consequently, in cultivars with smaller basal pores, self-pollination becomes less reliable, making these cultivars more prone to cross-pollination. This may present a limitation for both breeders and farmers, as the maintenance of self-pollination is important for preserving HS-tolerance traits across generations. A larger basal cleft has been associated with improved pollen deposition on the stigma (Matsui, 2003). Remarkably, experimental extension of the basal opening by 100 μm was reported to lower HS-induced sterility by 20% and to enhance heat tolerance by approximately 0.66 °C (Liu et al., 2023). Two quantitative trait loci (QTLs), *qBDL2-2* and *qBDL10* were associated with the control of basal dehiscence length in rice anthers under HS, and may be further explored to better understand and exploit this strategy for heat tolerance (Zhao et al., 2016). Thus, breeding rice genotypes with larger basal dehiscence may improve pollen shedding and partially offset the negative effects of high temperature on pollen swelling and theca dehiscence (Khan et al., 2019).

Tapetum development and its timely degeneration is key for anther dehiscence, being tightly coordinated with microspore maturation. Under normal conditions, the tapetum begins to degrade soon after the release of microspores from tetrads, and the middle layer undergoes programmed cell death (PCD) to enable anther dehiscence and the release of mature pollen. The anther is typically composed of 4 layers, epidermis, endothecium, middle cell layers and tapetum, and under normal conditions, the anther wall undergoes PCD to allow the release of mature pollen. However, exposure to high temperatures compromise dehiscence and, subsequent release of viable pollen, due to failures in the PCD process in the epidermal layer and excessive mitochondrial ROS production in the tapetum (Liu et al., 2023). Some thermosensitive genic male sterility (TGMS) genes highlight this vulnerability. *OSTMS15* (*O. sativa* ssp. *japonica* ZH11) encoding an LRR-RLK protein MULTIPLE SPOROCYTE1 is required for normal tapetum development (Han et al., 2023). Similarly, the rice TGMS line *ostms19* was shown to regulate pollen formation under HS conditions. Mutation of the pentatricopeptide repeat protein encoded by *OsTMS19* promotes excessive ROS accumulation in anthers during pollen mitosis under HS, ultimately resulting in pollen sterility (Zhou et al., 2024b). Notably, some tapetum-specific genes (*OsC6*, *OsRAFTIN*, *TDR*) remain stable under HS, suggesting selective sensitivity within tapetal regulatory networks. Although these genes are not directly involved in the HS response, they may still be useful as molecular markers of the anther or tapetum in studies of heat-induced injury (Endo et al., 2009).

A way to improve male HS tolerance is to enhance molecular/physiological defenses in the male structures. For instance, *OsLAC12* encoding a heat-induced laccase, contributes positively to rice tolerance to HS. At the heading stage, overexpression of *OsLAC12* enhanced the antioxidant enzyme system in spikelets, modulated ROS levels in anthers, and alleviated HS-induced impairment of pollen viability. By contrast, *oslac12* mutants exhibited an average reduction of 10.8% in pollen viability relative to the wild type, together with significantly decreased seed-setting rates (Ding et al., 2025). Similarly, *PROTEIN DISULFIDE-ISOMERASE 1 (OsPDIL1-1*) maintains ROS homeostasis during anther development by modulating NADPH oxidase activity. RNAi-mediated silencing of *OsPDIL1* ***-*** *1* leads to excessive ROS accumulation, severely reducing pollen viability and floret fertility under HS (Zhao et al., 2023b). Together, these findings highlight the critical role of antioxidant regulation in mitigating HS-induced damage in male reproductive structures. Phytohormones also play a crucial role in helping plants cope with HS. In rice, HS has been associated with increased abscisic acid (ABA) levels, which in turn trigger ROS accumulation and premature PCD, leading to abnormal degeneration of the tapetum and other inner layers. The ABA-activated protein kinase 2 (SAPK2) is involved in the regulation of tapetal PCD. Loss of *OsSAPK2* function in null mutants disrupts ABA signal transduction, leading to abnormal tapetum degeneration. Under HS, *sapk2* mutants show a stronger reduction in pollen viability, which has been associated with oxidative stress caused by ABA-mediated ROS accumulation in anthers (Zhao et al., 2023a). *9-CIS-EPOXYCAROTENOID DIOXYGENASE 1* (*OsNCED1*), which encodes a key enzyme in the ABA biosynthetic pathway, has been associated with improved heat tolerance in rice at the heading and flowering stages. Overexpression of *OsNCED1* increased pollen viability, seed-setting rate, and the activities of superoxide dismutase and peroxidase, suggesting that it enhances heat tolerance by strengthening antioxidant capacity (Zhou et al., 2022). Another phytohormone, salicylic acid (SA) was found to reduce ROS accumulation in anthers, thereby preventing tapetal PCD and degradation. In this process, *ETERNAL TAPETUM 1* (*EAT1*), *MICROSPORELESS 2* (*MIL2*), and *DEFECTIVE TAPETUM AND MEIOCYTES 1* (*DTM1*), genes associated with tapetum development, were reported to be involved in the SA-mediated prevention of heat-induced tapetum degradation (Feng et al., 2018). In parallel, localized increases in auxin (Indole-3-acetic acid, IAA) may delay dehiscence by inhibiting septum breakdown. The auxin biosynthesis genes, including members of the *YUCCA* family, are upregulated in response to exogenous application of synthetic auxin at the flowering stage, increasing the expression of *YUC1*, *YUC9*, *YUC11*, and other pistil-expressed genes, thereby promoting normal PT growth under HS (Zhang et al., 2018). These findings highlight that a finely balanced regulation of ROS homeostasis and hormone signalling is critical for anther development and dehiscence under HS and point to the importance of hormone management in improving crop fertility and stress resilience.

**FIGURE 3:**
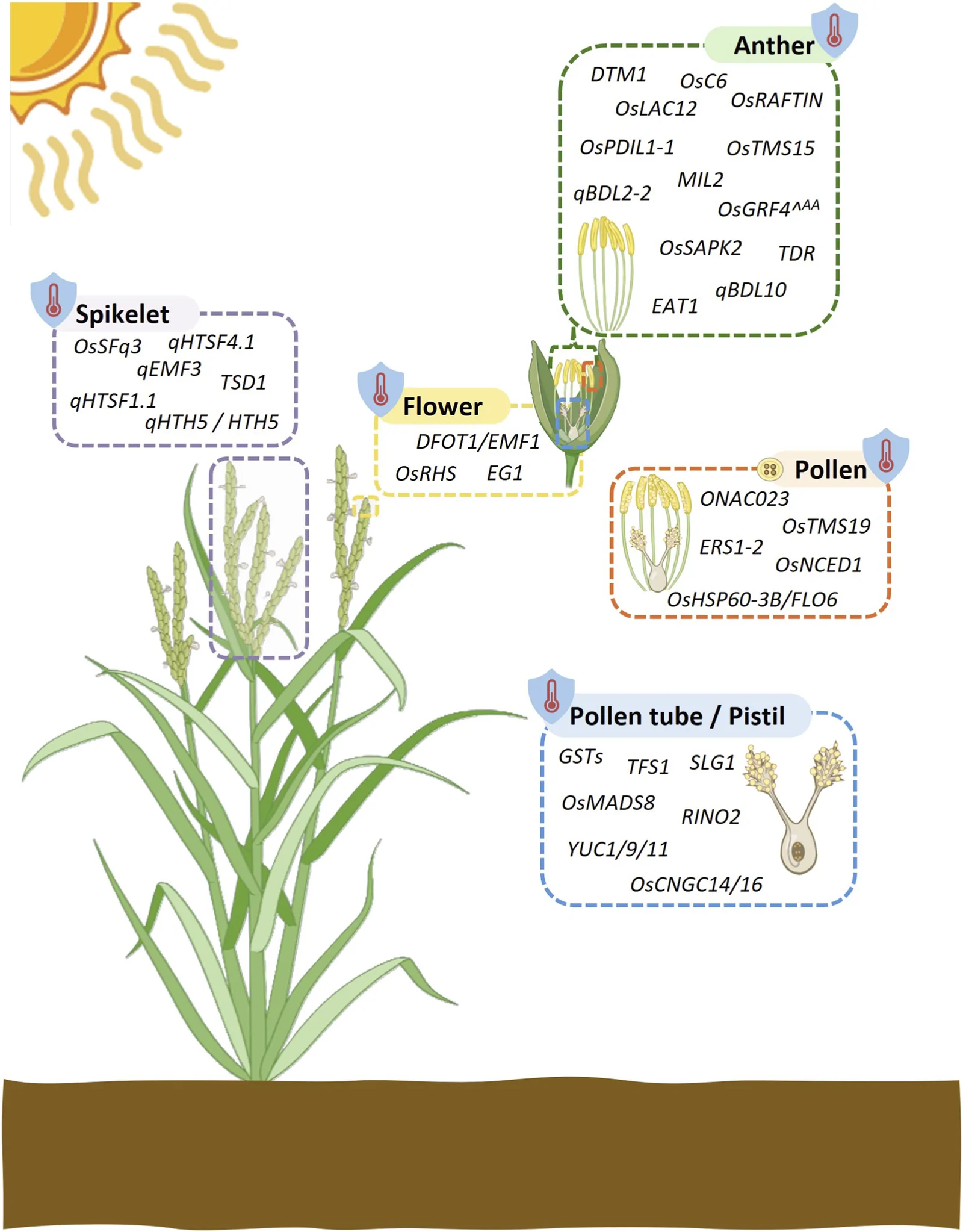
Schematic representation of stage-specific processes affected by elevated temperature in the anther, pollen, and pistil, during reproduction, highlighting the genes involved in these events.

Once PGs are ready to be released, anthesis, which is the most crucial and sensitive phase of rice reproduction, starts (Shrestha et al., 2022). The PGs must travel a short distance to reach their destination, the stigma surface, which in rice is bifurcated and feathery, facilitating pollen capture. The temperature continues to play an important role in pollen viability, hydration, germination, and subsequent growth of PTs. Exposure of pollen to HS during anthesis has been demonstrated to substantially compromise its viability, even after short periods of only a few minutes. Moreover, during anthesis, when spikelets are open, high temperatures rapidly disrupt pollen osmotic regulation, leading to decreased carbohydrate, protein, and vitamin C levels, which in turn inhibit PT growth on the stigma (Coast et al., 2016; Rieu et al., 2017; Shrestha et al., 2022). Interestingly, the naturally partial-opening of rice florets during anthesis can mitigate some of these negative effects. Cleistogamous spikelets, in which florets remain closed during anthesis, have been shown to maintain an internal environment approximately 1.8 °C cooler than ambient conditions, thereby improving seed set under HS above 38 °C (Koike et al., 2015; Liu et al., 2023). Given that heat-induced male sterility represents one of the major constraints limiting yield under HS conditions, understanding the genetic regulators controlling anther development, pollen viability and male reproductive processes is essential for improving crop productivity and quality. A comprehensive table of genes involved in rice reproductive responses to heat stress is compiled in Table 1, and a scheme illustrating their roles across the different reproductive stages is presented in Figure 3.

**TABLE 1:** List of genes involved in heat stress responses during rice reproduction.

QTL/Locus/Gene		Organ affected	Function/Validated role under HS	Reference
*DFOT1/EMF1*	*Diurnal Flower Opening Time 1/Early Morning Flowering 1*	Flower	Regulates early flower opening through interaction with pectin methylesterase family members, affecting pectin and cellulose synthesis in the lodicule cell wall. Altered activity promotes lodicule swelling, earlier glume opening, and escape from damaging daytime heat	Xu et al. (2022)
*DTM1*	*Defective Tapetum and Meiocytes 1*	Anther	Gene associated with tapetum development that participates in SA-mediated prevention of heat-induced tapetum degradation, contributing to protection of reproductive tissues under HS.	Feng et al. (2018)
*EAT1*	*Eternal Tapetum 1*	Anther	Tapetum-associated gene involved in SA-mediated protection against heat-induced tapetum degradation, helping reduce ROS accumulation and prevent premature tapetal PCD under HS.	Feng et al. (2018)
*E.G.,1*	*EXTRA GLUME1*	Flower	Maintains the expression of floral organ identity genes *OsMADS1*, *OsMADS6*, and *OsG1* through a high-temperature-mediated mitochondrial lipid pathway, thereby preserving floral organ homeostasis under HS.	Zhang C. et al., 2016
*ERS1-2*	*GLUTAMYL-TRNA SYNTHETASE 1–2*	Pollen	Gene required for normal pollen development and meiosis under HS. Mutation causes delayed pollen development, impaired antioxidant capacity, excessive ROS accumulation, and ultimately male infertility under high temperature	Liu et al. (2024)
*FLO6*	*FLOURY ENDOSPERM6*	Pollen	Key component of pollen starch granule formation that acts together with *OsHSP60-3B* under HS. Its stabilisation supports pollen starch biogenesis, limits anther ROS accumulation, and helps prevent pollen abortion under high temperature	Lin S et al.(2023)
*GSTs*	*Glutathione S-transferases*	Pollen/Pistil	Stress-responsive enzymes that contribute to heat tolerance during anthesis by supporting glutathione metabolism and ROS homeostasis. Their activity is associated with improved spikelet fertility, pollen germination, and PT growth, likely through proanthocyanidin and fructose related pathways that help prevent heat-induced pistil dysfunction	Xu et al. (2026)
*MIL2*	*Microsporeless 2*	Anther	Tapetum-associated gene involved in SA-mediated protection against heat-induced tapetum degradation, helping reduce ROS accumulation and maintain tapetum integrity under HS.	Feng et al. (2018)
*ONAC023*	*NAC domain-containing protein 23*	Pollen	Transcription factor associated with heat tolerance during the reproductive stage through regulation of pollen fertility under high temperature	Chang et al. (2024)
*OsC6* *OsRAFTIN* *TDR*	*ANTHER SPECIFIC PROTEIN 6 Tapetum Degeneration Retardation*	Anther	Tapetum-specific gene whose expression remains stable under high temperature. Although not directly involved in the HS response, it may serve as a molecular marker of anther/tapetum integrity in studies of heat-induced injury	Endo et al. (2009)
*OsCNGC14 OsCNGC16*	*CYCLIC NUCLEOTIDE-GATED CHANNEL 14 CYCLIC NUCLEOTIDE-GATED CHANNEL 16*	Pollen/Pistil	Ca^2+^-permeable cyclic nucleotide-gated channel involved in Ca^2+^ homeostasis. Contributes to heat tolerance by supporting PT growth under high temperature	Cui et al. (2020)
*OsEDS1*	*ENHANCED DISEASE SUSCEPTIBILITY 1*	Seed setting/grain yield	Positive regulator of rice thermotolerance that enhances catalase-mediated H_2_O_2_ scavenging under HS. Overexpression improves reproductive-stage performance, increasing seed setting, grain weight, and overall yield under high temperature	Liao et al. (2023)
*OsGRF4^AA^*	*GROWTH-REGULATING FACTOR 4*	Anther	Variant of *OsGRF4* that escapes miR396-mediated mRNA degradation and affects transcriptional and splicing regulation under HS. It alters the expression of genes involved in carbohydrate metabolism, chloroplast development, photosynthesis, lipid metabolism, and male fertility, ultimately reducing heat tolerance in rice anthers	Mo et al. (2023)
*OsHSP60-3B*	*HEAT SHOCK PROTEIN60-3B*	Pollen	Heat-induced plastid-localised chaperonin that positively regulates pollen thermotolerance. It interacts with and stabilises FLO6 under high temperature, thereby maintaining pollen starch granule biogenesis, reducing ROS accumulation in anthers, preventing cell death and pollen abortion, and ensuring normal male gametophyte development. Loss of function causes temperature-dependent male sterility, whereas overexpression enhances heat tolerance	Lin Y et al.(2023)
*OsLAC12*	*-*	Anther	Heat-induced laccase that positively regulates rice heat tolerance by enhancing the antioxidant enzyme system in spikelets, modulating ROS levels in anthers, and alleviating heat-induced reductions in pollen viability. Overexpression improves tolerance, whereas *oslac12* mutants show reduced pollen viability and significantly lower seed-setting rates	Ding et al. (2025)
*OsMADS8*	*-*	Pistil/Ovule	Essential for female reproductive development under high temperature, particularly pistil development. Knockout mutants show defects in ovule initiation, with some pistils lacking ovules, ultimately leading to failed fertilization	Shen et al. (2023)
*OsNCED1*	*9-CIS-EPOXYCAROTENOID DIOXYGENASE 1*	Pollen	Encodes a key enzyme in the ABA biosynthetic pathway and positively regulates heat tolerance in rice. Overexpression increases pollen viability, seed-setting rate, and superoxide dismutase and peroxidase activities, indicating enhanced antioxidant capacity under HS.	Zhou et al. (2022)
*OsPDIL1-1*	*PROTEIN DISULFIDE-ISOMERASE 1*	Anther	Protein disulfide isomerase-like protein that helps maintain ROS homeostasis during anther development by modulating NADPH oxidase activity. RNAi silencing increases ROS accumulation and severely reduces pollen viability and floret fertility under high temperature	Zhao et al. (2023b)
*OsRHS*	*Reduced Heat Stress Tolerance 1*	Flower	Cytoplasm-localised protein that acts as a negative regulator of heat tolerance during the flowering stage, at least partly by repressing stress-related gene expression under HS. Overexpression reduces heat tolerance, whereas RNAi silencing or knockout enhances tolerance without yield penalty	Mao et al., 2025
*OsSAPK2*	*ABA-activated protein kinase 2*	Anther	Stress-activated protein kinase involved in ABA signal transduction and regulation of tapetal PCD. Loss of function disrupts normal tapetum degeneration and, under HS, leads to greater reductions in pollen viability associated with oxidative stress and ABA-mediated ROS accumulation in anthers	Zhao et al. (2023a)
*OsSFq3*	*-*	Spikelet	Gene highly homologous to FLO6 that contributes to spikelet fertility and grain quality under high temperature. It participates in glycometabolism and sugar homeostasis, supporting tolerance to HS and making it a promising target for breeding	Park et al. (2021)
*OsTMS15*	*Thermosensitive genic male sterility 15*	Anther	Encodes the LRR-RLK protein MULTIPLE SPOROCYTE1, which is required for normal tapetum development during pollen formation.A point mutation impairs tapetal function under HS and leads to male infertility	Han et al. (2023)
*OsTMS19*	*Thermosensitive genic male sterility 19*	Pollen	Encodes a pentatricopeptide repeat protein required for pollen formation under HS. Mutation promotes excessive ROS accumulation in anthers during pollen mitosis, ultimately causing pollen sterility	Zhou et al. (2024b)
*qBDL2-2* *qBDL10*	*-*	Anther	QTL associated with the control of basal dehiscence length in rice anthers under high temperature, suggesting a role in anther opening and heat tolerance	Zhao et al. (2016)
*qEMF3*	*-*	Spikelet	QTL associated with the early-morning flowering trait that advances flower opening by about 1.5–2 h, enabling spikelets to escape damaging high temperatures during anthesis	Hirabayashi et al. (2014)
*qHTH5/HTH5*	*-*	Heading stage/Spikelet	QTL on chromosome 5 associated with heat tolerance at the heading stage. HTH5 is predicted to encode a pyridoxal phosphate homeostasis protein that reduces ROS accumulation under HS. Overexpression improves seed-setting under HS, whereas suppression increases susceptibility; promoter variation is associated with expression differences and heat-tolerance diversity	Cao et al. (2022)
*qHTSF1.1*	*-*	Spikelet	QTL associated with reproductive-stage thermotolerance that contributes to the maintenance of spikelet fertility under high-temperature conditions	Ye et al. (2012)
*qHTSF4.1*	*-*	Spikelet	Validated QTL for reproductive-stage heat tolerance that enhances spikelet fertility under temperatures above 37 °C. Increased fertility by about 15% in a near-IR64 backcross population	Ye et al. (2015)
*RINO2*	*Rice myo-inositol-3-phosphate synthase 2*	Pollen/Pistil	Regulates Ca^2+^ signalling and actin filament organisation in an inositol-dependent manner. *rino2* mutants show increased susceptibility of spikelet fertility to heat injury at anthesis due to stronger impairment of pollen germination and PT growth in the pistil, indicating that *RINO2* is required to maintain spikelet fertility under HS.	Zhou et al. (2024a)
*SLG1*	*Slender Guy 1*	Pollen/Pistil	Recessive tRNA-related mutation that affects protein homeostasis during the stress response. Under HS, it is associated with reduced pollen fertility and impaired PT elongation	Xu et al. (2020)
*TFS1*	*Thermo-sensitive Female Sterility 1*	Pollen/Pistil	Encodes ARGONAUTE7 (AGO7) and functions as a thermo-sensitive female fertility regulator. A point mutation disrupts the miR390–TAS3–ARF small RNA pathway, impairing PT growth under high temperature and causing female infertility	Li et al. (2022)
*TSD1*	*Thermo-Sensitive Spikelet Defects 1*	Spikelet	Transcription factor that physically interacts with a YABBY protein to regulate spikelet morphogenesis in response to high temperature	Cai et al. (2023)
*YUC1 YUC9 YUC11*	*-*	Pistil	Auxin biosynthesis gene whose expression is upregulated in the pistil following exogenous auxin application at the flowering stage, supporting normal PT growth under HS.	Zhang et al. (2018)

## Pollen-pistil interactions under heat stress

Successful PT growth from the stigma to the ovule depends on the pollen - stigma interaction, which includes pollen adherence, hydration, and germination (Dresselhaus and Franklin-Tong, 2013). Upon deposition on the stigma and recognition, PGs quickly take up water and activate their metabolic processes, an event referred to as pollen hydration. This process relies heavily on the integrity of the pollen wall, particularly the innermost plasma membrane layer, which is rich in proteins and lipids and is essential for water uptake (Edlund, 2004). Exposure to HS can compromise membrane stability, thereby disrupting pollen hydration (Das et al., 2014). At the structural level, HS alters the composition and degree of unsaturation of phospholipids in pollen membranes, reducing membrane fluidity and compromising viability. Furthermore, the interaction of pollen with the stigma may also be affected due to possible abnormal patterns in the PG wall (Liu et al., 2023).

Once recognized as compatible, the PG is hydrated and begins to germinate. During germination, Calcium (Ca^2+^) plays a central role in establishing the tip-focused gradient required for PT growth. Proteins such as cyclic nucleotide-gated channels (CNGCs) and annexins modulate Ca^2+^ permeability and signaling across cell membranes (Gao et al., 2016). In rice, *OsCNGC14* and *OsCNGC16* have been identified as key genes contributing to HS tolerance, maintaining Ca^2+^ homeostasis and promoting PT growth under HS (Cui et al., 2020). In parallel, mature PGs function as carbohydrate reservoirs that support germination and PT growth. However, episodes of HS reduce carbohydrate accumulation and inhibit sucrose synthase activity, thereby limiting the energy supply required for successful fertilization (Begcy et al., 2019). Indeed, in several different rice cutlivars, more than half of the PGs did not germinate when the temperature was between 30 °C–35 °C (Coast et al., 2016).

At the molecular level, some studies demonstrated multiple genes which coordinate pollen thermotolerance. *SLENDER GUY 1* (*SLG1*) encodes the cytosolic tRNA 2-thiolation protein (RCTU2) and plays a key role in rice HS response. A recessive mutation affecting tRNA thiolation disrupts protein homeostasis under stress. Under HS conditions, this impairment leads to reduced pollen fertility and defective PT elongation (Xu et al., 2020). Another gene, *RICE MYO-INOSITOL-3-PHOSPHATE SYNTHASE 2* (*RINO2*) regulates Ca^2+^ signalling and actin filament organisation in an inositol-dependent manner. In *rino2* mutants it was observed an increased susceptibility of rice spikelet fertility to heat injury at anthesis mainly due to a severe impairment of pollen germination and PT growth in the pistil. These findings indicate that *RINO2* is required for the heat-tolerance-related regulation of pollen germination and PT growth in rice, thereby contributing to the maintenance of spikelet fertility under HS (Zhou et al., 2024a). *ENHANCED DISEASE SUSCEPTIBILITY 1* (*OsEDS1*) acts as a positive regulator of rice thermotolerance by enhancing reproductive-stage performance under HS through the promotion of catalase-mediated H_2_O_2_ scavenging. This has been observed in *OsEDS1* overexpression lines where heat tolerance during the reproductive stage were markedly improved, due to substantial increases in seed setting, grain weight, and plant yield (Liao et al., 2023). A recent study showed that glutathione S-transferases (GSTs), stress-responsive enzymes, appear to contribute to heat tolerance during anthesis. In two rice varieties exposed to HS, transcriptomic and physiological analyses indicated that GSTs, glutathione (GSH), proanthocyanidins (PAs), and fructose play important roles in maintaining ROS homeostasis and thereby in determining varietal differences in heat tolerance. Consistent with this, exogenous GST enhancers improved spikelet fertility, GST activity, pollen germination, and PT growth, suggesting that GST-mediated GSH metabolism helps prevent heat-induced pistil dysfunction through PA- and fructose-related pathways (Xu et al., 2026).

Importantly, the inhibition of PT growth under high temperatures is not solely attributable to the male gametophyte, the pistil also plays a critical role by providing essential resources and signaling cues for successful PT growth and elongation (Yao et al., 2024).

## Impact of heat stress on female reproductive development

Spikelet fertility is determined by the joint contribution of pollen viability and pistil functionality. Notably, Miura et al. (2011) demonstrated that pistils remained receptive through hand pollination even after a 5-day exposure to 41 °C, highlighting the higher thermotolerance of the pistil comparing to the particular vulnerability of stamens to elevated temperatures. However, studies across multiple crops demonstrate that female gametophytes are far from immune. In wheat *(Triticum aestivum* L.), abnormal embryo sac development was identified as the main cause of reduced seed set, even when pollination was performed with non-stressed pollen (Saini et al., 1983). Comparable reductions in female organ viability under HS have been reported in canola (*Brassica napus*; Polowick and Sawhney, 1988), sorghum (*Sorghum bicolor*; (Djanaguiraman et al., 2018b), and pearl millet (*Pennisetum glaucum*; (Djanaguiraman et al., 2018a). In rice, early studies suggested that pistils were relatively resilient. Satake and Yoshida (1978) reported that stigma receptivity remained unaffected even at 41 °C, while pollen sterility became significant above 35 °C. Endo (2009) also found that pistils subjected to HS during the early microspore stage retained their capacity to receive non-stressed pollen. However, these assessments were limited to surface-level processes such as stigma function and did not consider deeper developmental stages, including ovule and embryo sac formation, which determine the number of produced seeds. More recent investigations demonstrated that HS induces substantial structural and functional abnormalities within the female gametophyte (Lohani et al., 2020; Wang et al., 2021). Detailed anatomical analyses of contrasting rice genotypes highlight striking differences in pistil responses to HS. In the heat-sensitive cultivar IR64, exposure to 38 °C–40 °C during gametogenesis resulted in degeneration of all four megaspores immediately after meiosis, rather than the normal degeneration of three. Additional abnormalities included missing or mispositioned nuclei, degenerated egg apparatus, and, in extreme cases, complete ovary collapse. At the heading stage under control conditions (30 °C), embryo sacs typically contained one egg cell and two polar nuclei positioned correctly for subsequent embryo and endosperm development. Under HS, however, IR64 exhibited a high frequency of embryo sacs that were completely degenerated, lacked the egg cell and/or polar nuclei, or showed abnormal nuclear positioning. By contrast, the tolerant genotype Nagina 22 (N22) maintained more than 94% normal embryo sacs under similar stress, while IR64 dropped to around 82%–84% at 40 °C. These abnormalities in IR64 pistils directly correlated with significant declines in spikelet fertility, even when pollinated with viable, non-stressed pollen (Shi et al., 2022). Such findings in rice, parallel observations in other species. In tomato, HS led to degeneration of the egg cell and the synergids (Iwahori, 1966), while in cotton, egg cell and synergid differentiation was impaired (Snider et al., 2009). In wheat, altered polarity of nuclei within the embryo sac disrupted development, leading to reduced fertility (Saini et al., 1983). The distortion of polarity in rice embryo sacs, caused by irregular nuclear positioning under HS, suggests a similar mechanism (Zeng et al., 2009). In addition to structural defects, HS imposes strong biochemical and metabolic stress on the pistil. Normal female gametophyte development depends on finely balanced levels of ROS and antioxidant enzyme activity, which also govern pollen-pistil interactions during fertilisation (Sharma and Bhatla, 2013; Zinta et al., 2016). Combined heat and drought stress induces extensive metabolic and transcriptomic reprogramming in both anthers and pistils, with disruptions observed in carbohydrate metabolism, the tricarboxylic acid cycle, and amino acid pathways (Li et al., 2015). These changes likely exacerbate embryo sac abortion by limiting the energy and signalling resources necessary for fertilization and early seed development. Moreover, in several different species, PT growth rates decrease under HS because of the pistil’s decreased soluble carbohydrate content (Snider et al., 2011). Not only carbohydrate content is important in the pistil, but also the content of auxins, flavonols and ROS homeostasis, playing an important role in germination and PT elongation in rice (Shrestha et al., 2022; Yao et al., 2024). Zhang et al. (2018), demonstrated that, in rice pistils of heat-sensitive genotypes, exposure to HS led to a reduction in auxin levels, which consequently impaired the rate of PT growth.

Only a few genes have been linked so far to female reproductive heat tolerance in rice. A *THERMO-SENSITIVE FEMALE STERILITY 1* (*TFS1*), which encodes ARGONAUTE7 (AGO7), is a thermo-sensitive female fertility regulator in rice. A point mutation in *TFS1* disrupts AGO7-mediated small RNA function in the miR390–TAS3–ARF module, thereby impairing PT growth under high temperature and causing female infertility (Li et al., 2022). *OsMADS8* is essential for female reproductive development under HS conditions, particularly for rice pistil development. It has been shown that *osmads8* knockout mutants exhibit defects in ovule initiation at high temperature, with several pistils lacking ovules, ultimately leading to failed fertilization (Shen et al., 2023). *OsSFq3*, a *FLO6* homolog, supports spikelet fertility and grain quality under high temperatures by maintaining sugar homeostasis and glycometabolism, representing a promising target for breeding heat-tolerant varieties (Park et al., 2021). Overall, the evidence shows that while male gametophyte sterility is a dominant factor determining HS sensitivity during the reproductive phase, abnormalities in the female gametophyte also play a significant role in spikelet fertility loss. Embryo sac degeneration, mispositioned nuclei, ovary collapse, sugar starvation, and oxidative damage all contribute to reduced reproductive success, particularly in sensitive genotypes such as IR64. By contrast, tolerant cultivars like N22 maintain more stable embryo sac structures, stronger antioxidant defences, and better carbohydrate allocation, enabling them to sustain fertility under elevated temperatures. Using transcriptomic and metabolomic analyses, some genes, key metabolites, and novel pathways closely associated with rice heat perception, signal transduction, HS response, and heat tolerance have been identified, providing potential molecular markers and target genes for the development of heat-tolerant crop varieties (Guan et al., 2025).

## Rice reproduction: mechanisms of heat tolerance and heat avoidance

Heat tolerance (thermotolerance) and heat avoidance are generally considered conceptually distinct strategies. Nevertheless, they are not mutually exclusive, may overlap within the same plant response, and together form part of heat resistance (Khan et al., 2019). Heat tolerance is defined as the capacity of a plant to sustain normal growth and maintain economic yield under high-temperature conditions through structural or metabolic adjustment (Yamanouchi et al., 2002; Hasanuzzaman et al., 2013). This trait is highly specific, and marked differences may be observed not only among closely related species, but also among different organs and tissues of the same plant. To withstand elevated temperatures, plants have evolved a range of adaptive mechanisms. Heat avoidance includes survival strategies based on long-term phenological and morphological adaptations, as well as short-term avoidance or acclimation responses, such as modifications in leaf structural composition, orientation, shape, and size (Fitter and Hay, 2002), transpirational cooling, changes in membrane lipid composition, and the completion of reproduction before the onset of HS (Julia and Dingkuhn, 2012), by changing their HS resistance depending on the year station (Hasanuzzaman et al., 2013). Moreover, heat avoidance may also involve crop management practices. In rice, for example, early sowing, adjustments to site-specific cropping systems and irrigation practices, and the adoption of early- or late-maturing cultivars to avoid exposure to high temperatures during grain filling are strategies that can be applied to cope with HS (Oh-e et al., 2007; Krishnan et al., 2011).

There are many mechanisms for rice to avoid HS, for example, by changing the plant architecture (to protect the panicles), adjusting the time of panicle emergence or lowering panicle temperature by transpiration cooling (Julia and Dingkuhn, 2012; Xiong et al., 2015). It was shown that the damage by HS is more severe in panicles than to leaves due to the transpiration rate of the flag leaf since its rate is higher than that of the spikelet under HS, which results in lower leaf temperatures relative to those of the spikelets (Zhang C. et al., 2016). Accordingly, one way to avoid this is the development and selection of genotypes with a suitable plant architecture that may confer greater tolerance to high temperature. For instance, genotypes where their panicles are surrounded by bigger and greater number of leaves, it will create a sun shield to the anthers and a higher transpiration rate which promotes transpirational cooling (decreasing panicle temperature) and reduce the anther water loss maintaining the spikelet fertility (Shah et al., 2011). Reduced water loss from the anther helps to maintain PG swelling, which is essential for anther dehiscence (Khan et al., 2019). This occurs in N22 cultivar where in HS conditions their height was higher, resulting in increased thermotolerance (Poli et al., 2013). Also, varieties with asynchronous tillers and panicle development have been reported to exhibit lower yield losses and greater tolerance to HS at critical developmental stages, including the reproductive stage, since not all panicles are exposed to high temperature simultaneously (Khan et al., 2019). Although transpirational cooling can protect reproductive structures and improve spikelet fertility, its effectiveness is reduced in regions with high atmospheric humidity (Khan et al., 2019). Likewise, although asynchronous panicle development may theoretically reduce heat damage, in practice it can result in longer ripening periods, which may be undesirable for both farmers and breeders. Despite all of these, further approaches are still needed to better translate this knowledge into rice improvement strategies.

Other strategy for rice to escape or avoid HS is adjusting the time of spikelet or flower opening to where the air temperature is cooler (Khan et al., 2019). Spikelet sterility is among the most measured traits used to assess heat tolerance, and variation among rice genotypes in their ability to prevent or reduce it may be considered a tolerance mechanism (Jagadish et al., 2007; Julia and Dingkuhn, 2012). This phenomenon happens mainly due to the lower number of germinating PGs and impaired anther dehiscent under HS during anthesis (Matsui, 2003). As mentioned in the previous sections, timing during the reproductive stage, particularly at anthesis, is crucial for successful fertilization. Several studies have shown that exposure to high temperature for only 1 hour, or even less, is sufficient to induce sterility, whereas heat treatment applied 1 hour after fertilization does not cause sterility (Jagadish et al., 2007; Zhang C. et al., 2016). This indicates that spikelets become substantially more tolerant to HS once fertilization has occurred (Satake and Yoshida, 1978). Genotypic variation in anthesis timing exists among rice cultivars, with different cultivars showing distinct patterns of flower opening. Although flower opening time is under genetic control, it is also influenced by environmental factors such as air temperature, humidity, and solar radiation. Therefore, being a genetically regulated trait, it is possible to identify the genes and QTLs involved in its control, thereby improving our understanding of the mechanisms underlying heat tolerance. This trait could also be exploited in breeding programmes to enhance heat tolerance in rice genotypes grown under flooded rice systems (Sheehy et al., 2005; Bheemanahalli et al., 2017). A well-characterised QTL associated with the early-morning flowering trait in rice is designated *qEMF3*, which advances flower opening by approximately 1.5–2 h in cv. Nanjing 11 under temperate conditions in Japan and in cv. IR64 under tropical conditions in the Philippines, thereby allowing spikelets to escape damaging temperatures during anthesis (Hirabayashi et al., 2015). Moreover, additional QTLs and genes have been linked to heat tolerance in rice spikelets. Among them, *qHTSF4.1*, a validated QTL for heat tolerance at the flowering stage that enhances spikelet fertility under temperatures above 37 °C. In a backcross population with a near-IR64 background, *qHTSF4.1* increased spikelet fertility by about 15%, supporting its relevance for breeding heat-tolerant rice (Ye et al., 2015). *qHTSF1.1*, other QTL that was related to thermotolerance in the reproductive-stage in rice. Other study showed that both *qHTSF1.1* and *qHTSF4.1* explained a significant proportion of the variation in spikelet fertility under high-temperature conditions, accounting for 12.6% and 17.6% of the phenotypic variation, respectively, and are therefore valuable targets for breeding programmes (Ye et al., 2012). qHTH5 is a QTL located on chromosome five associated with heat tolerance at the heading stage, which marks the transition from the vegetative to the reproductive phase. Overexpression of *HTH5* increased the seed-setting rate of rice plants under HS, whereas suppression of *HTH5* resulted in greater susceptibility to HS. In addition, variation in the promoter region of *HTH5* is associated with differences in its expression level and with diversity in heat tolerance, highlighting this gene as a promising target for improving rice thermotolerance (Cao et al., 2022).

Some genes involved in the morphogenesis of rice floral organs have already been identified and may be of particular interest in the context of HS (Xing et al., 2024). *E.G.,1* (*EXTRA GLUME1*) has been reported to maintain the expression of the floral organ identity genes *OsMADS1*, *OsMADS6*, and *OsG1* through a high-temperature-mediated mitochondrial lipid pathway, thereby contributing to floral organ homeostasis (Zhang B. et al., 2016). In addition, *TSD1 (THERMO-SENSITIVE SPIKELET DEFECTS 1)*, which functions as a transcription factor, physically interacts with a YABBY protein to regulate spikelet morphogenesis in response to high temperature (Cai et al., 2023). In relation to early flower opening, *DFOT1*/*EMF1 (DIURNAL FLOWER OPENING TIME 1/EARLY MORNING FLOWERING 1*), interacts with several members of the pectin methylesterase family thereby influencing pectin and cellulose synthesis in the lodicule cell wall. It was observed that in the *dfot1* mutant, reduced PME activity is associated with increased pectin methylation, which promotes water uptake by the lodicule cell wall and enhances lodicule swelling. This increased swelling pushes the glumes apart, resulting in earlier flower opening and thereby allowing escape from high daytime temperatures (Xu et al., 2022).

Advances in genomic technologies, including long-read sequencing and novel scaffolding strategies, have greatly expanded chromosome-scale assemblies and revealed new molecular components of HS responses (Sun et al., 2022). However, as research progresses and these systems are examined in greater detail, additional unknowns continue to emerge. This is particularly evident in the study of HS during plant reproduction, which, as mentioned previously, is of critical importance for both food security and plant survival. In rice, the second specie to have its whole genome sequenced and assembled (Sun et al., 2022), numerous molecular mechanisms underlying the HS response have been uncovered. Nevertheless, many of these mechanisms remain to be characterised, and elucidating them is essential to understand how plants respond and adapt to HS and how can this be applied to benefit the general population (Ohama et al., 2017). However, before exploring some of the key players identified in recent years, it is first necessary to establish the basis of HS response mechanisms and clarify the concepts of heat tolerance and heat avoidance.

## Research gaps in understanding rice reproduction under heat stress

**FIGURE 4:**
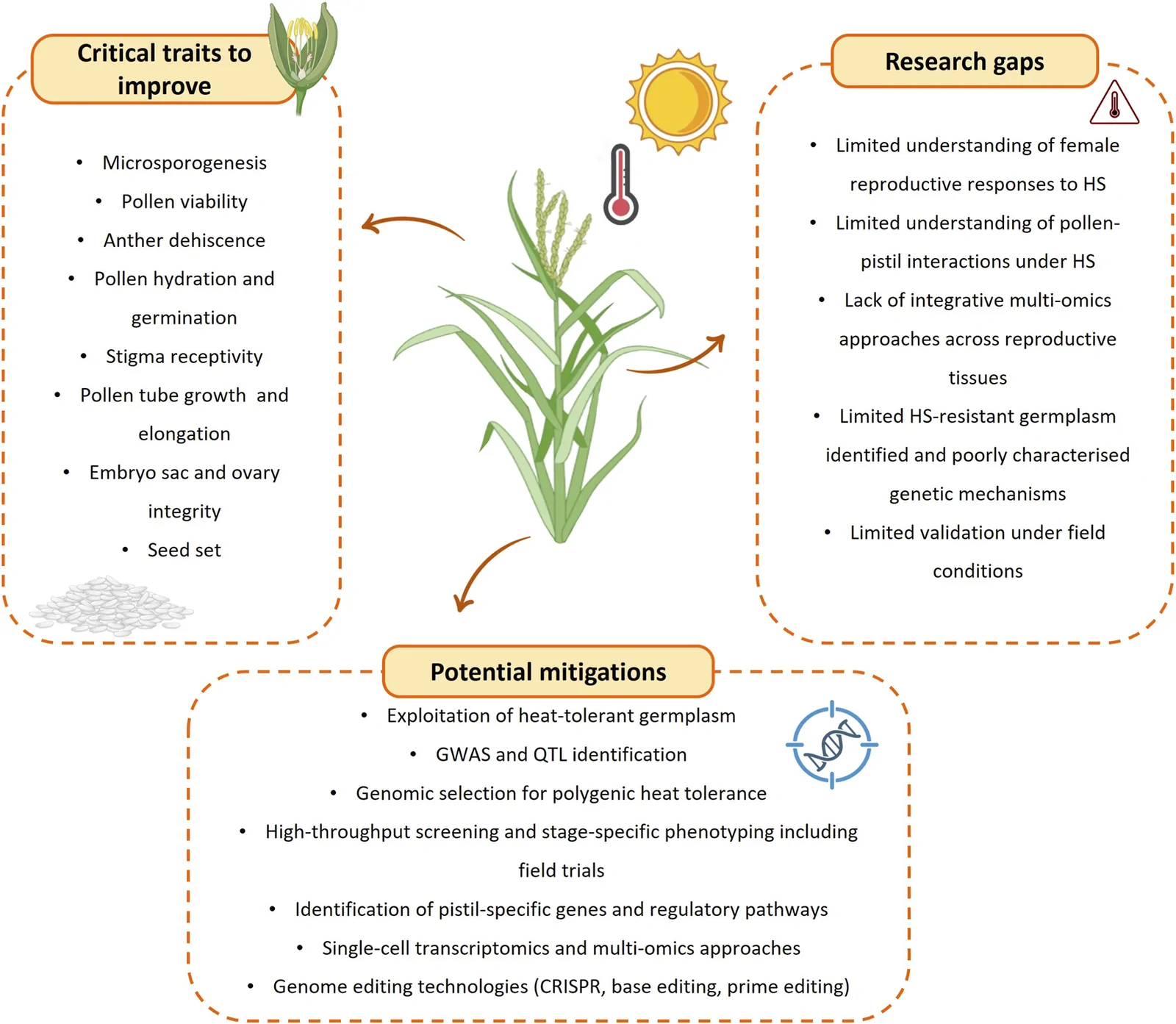
Schematic overview of the main challenges and opportunities for improving rice resilience to heat stress during reproductive development. The left panel highlights key reproductive and yield-related traits that should be prioritized in breeding programs to enhance heat tolerance. The right panel summarizes major research gaps that limit our understanding of heat stress responses and their application to crop improvement. The bottom panel outlines potential mitigation strategies, including genetic improvement, molecular approaches, and agronomic measures, to enhance crop resilience and sustain grain yield under increasing temperatures.

Although knowledge of rice responses to HS has advanced, research on reproductive processes has significant gaps. Most studies have focused on male organs, with an emphasis on pollen viability, germination, and anther dehiscence (Jagadish et al., 2021). On the other hand, the pistil, ovules, and embryo sac remain largely understudied under HS, despite their central role in fertilisation and seed production. A complex yet well-coordinated regulatory network comprising genes, metabolites, signalling molecules, and hormones is essential for female gametophyte development, pollen-pistil interactions, fertilization, and early embryogenesis, and HS has a major impact on each of these processes. Nonetheless, most of the reviews emphasise that reproductive failure is often attributed to ‘pollen sterility’, while the contribution of female reproductive organs remains a neglected dimension of HS tolerance (Wang et al., 2021). This bias represents a critical knowledge gap that must be addressed to achieve a complete understanding of reproductive resilience under CC. In addition, little information exists on pistil-specific molecular responses to HS, such as transcriptional reprogramming, ROS homeostasis, hormonal signaling, and pollen–pistil interactions (Wang et al., 2021). Most studies so far have looked at only one developmental stage or a single omics layer. It is still unclear how rice plants adapt to HS dynamically during the entire reproductive cycle, from panicle development to flowering and grain filling (Guan et al., 2025). The lack of field-realistic studies further limits the translation of laboratory findings into breeding practice. Most experiments are performed under constant high-temperature regimes, whereas actual field conditions involve rapid fluctuations, heat spikes, and elevated night temperatures, often combined with drought or humidity stress. High night temperatures, for instance, have been shown to significantly reduce spikelet fertility and grain quality, yet the underlying mechanisms remain poorly understood (Wang et al., 2021; Shi et al., 2022). Moreover, pollen–pistil interactions under field-like conditions remain largely unexplored in most crops (Figure 4), despite their importance for understanding how realistic temperature fluctuations can affect the molecular signalling pathways involved in these processes.

## From breeding solutions to genome editing technologies

Breeding techniques ranging from conventional, speed breeding to molecular-assisted approaches continue to be the primary strategy among the various approaches to reduce the impact of HS on rice, but are time-consuming solutions (Yiwei et al., 2024). The exploitation of heat-tolerant germplasm represents a critical foundation for genetic improvement. Traditional varieties, such as N22, aus-type, and wild Oryza landraces, have been widely used as donors of heat tolerance, particularly for maintaining spikelet fertility under high-temperature conditions (Jagadish et al., 2010; Poli et al., 2013; Prasanth et al., 2017). The use of diversity panels, combined with genome-wide association studies (GWAS), has enabled the identification of QTLs associated with reproductive-stage heat tolerance, although their validation under field conditions remains limited (Zhao et al., 2011; Lafarge et al., 2017).

Despite these, data on HS-resistant rice varieties and the genetic mechanisms underlying tolerance, particularly during the reproductive phase, are still limited, which impacts existing breeding programmes (Visakh et al., 2024). Addressing the knowledge gaps identified in this review will therefore have direct implications for breeding efficiency. For instance, if breeders focus only on screening pollen viability under HS, they may inadvertently discard genotypes with strong pistil tolerance. A better understanding of female organ resilience is thus essential to ensure that selection strategies capture the full potential for reproductive success.

GWAS have identified multiple single nucleotide polymorphisms (SNPs) and haplotypes associated with reproductive heat tolerance, including loci controlling pollen viability, anther dehiscence, and spikelet fertility under combined HS-drought (Lafarge et al., 2017; Li et al., 2023). These GWAS-derived markers enable high-throughput screening across diverse germplasm collections. Identifying pistil-specific genes and QTLs can provide novel targets for marker-assisted selection (MAS) and genome editing (Lin Y et al,.2023). QTLs mapping and GWAS convergence has pinpointed stable regions like *qHTSF4.1* (chromosome 4) and *qHTSF6.1* (chromosome 6) controlling anther filament elongation and pistil resilience (Ye et al., 2015; Lafarge et al., 2017). However, the polygenic nature of reproductive heat tolerance often limits the efficiency of MAS when used alone (Collard and Mackill, 2008). In this context, genomic selection (GS), which captures genome-wide small-effect loci, represents a promising complementary strategy, particularly when combined with high-throughput and stage-specific phenotyping (Spindel et al., 2015). Moreover, understanding reproductive stage-specific vulnerabilities will enable breeders to design precise phenotyping assays that target the most heat-sensitive phases, improving the accuracy of selection in breeding programs (Nalini Chandran et al., 2024). Recent advances have shown that integrating GWAS with tissue-specific transcriptomic and functional genomic datasets will improve the identification of candidate genes underlying complex reproductive traits. For example, Ming et al. (2023) combined GWAS with population-level transcriptomic data from 1 to 2 mm young rice panicles, a developmental stage critical for panicle architecture establishment, and performed transcriptome-wide association analyses to identify genes whose expression was associated with panicle traits. Because conventional GWAS based solely on marker–trait associations may have limited power to resolve causal genes, particularly for traits controlled by multiple small-effect loci, the authors further decomposed gene expression into cis- and trans-regulated components. This approach allowed them to prioritize putative causal genes and regulatory networks associated with the number of spikelets per panicle (SPP), identifying 36 candidate causal genes, including *SDT/MIR156j* and Os*MADS17*. Importantly, Os*MADS17* was experimentally validated as a negative regulator of SPP that modulates *SDT* expression, highlighting the value of integrating genetic variation, tissue-specific expression data and functional validation to uncover regulatory mechanisms controlling rice reproductive architecture. Recent studies further support the need to integrate genetic association mapping with functional genomic information to dissect complex rice reproductive traits. Kordi et al. (2025) performed a GWAS for 20 rice panicle architecture traits and identified multiple QTLs distributed across the 12 chromosomes. By defining QTL windows according to chromosome-specific linkage disequilibrium decay and combining GWAS results with publicly available panicle-versus-stem RNA-seq data, protein–protein interaction network analysis, haplotype analysis and epistasis testing, the authors prioritized several candidate genes and gene families potentially involved in panicle development, including regulators related to transcriptional control, ubiquitination, protein kinases, cytoskeleton dynamics and brassinosteroid signalling. Although this study does not provide the same level of causal resolution as the approach proposed by Ming et al. (2023), it reinforces the view that rice panicle architecture is controlled by multiple loci and regulatory pathways, and that post-GWAS integration with tissue-relevant expression and network data will be key to improve the prioritization of candidate genes for reproductive traits. More recently, GWAS-based studies have further reinforced the value of integrating association mapping with functional genomic evidence to refine candidate gene selection for rice breeding. For example, Singh et al. (2026) identified a pleiotropic QTL region on chromosome seven associated with panicle weight and panicle length, and proposed *Os07g0112700*/*OsENODL19*, which encodes a cupredoxin domain-containing protein, as a candidate gene linked to this marker–trait association. The use of RiceXPro and publicly available RNA-seq datasets showed that *Os07g0112700* is preferentially expressed in reproductive organs and early grain, embryo and endosperm developmental stages, supporting its possible involvement in reproductive and yield-related traits.

These studies show the potential of moving beyond marker-trait associations alone towards integrative approaches incorporating tissue-specific expression, regulatory information and functional validation. However, while such strategies have been applied to panicle or inflorescence architecture, their application to more specific reproductive structures and processes is still limited. Several transcriptomic resources have been generated for rice reproductive development, including expression atlases covering multiple reproductive stages, anther-specific co-expression networks, and transcriptomic datasets from stigma, ovary and embryo sac tissues (Fujita et al., 2010; Kubo et al., 2013; Wu et al., 2015; Lin et al., 2017; Yu et al., 2017). These resources have revealed tissue-enriched genes and candidate regulators involved in anther and pollen development, female gametophyte development, pollen-pistil interactions and early seed formation. The integration of these resources with population-level GWAS/TWAS frameworks to resolve the genetic and regulatory basis of reproductive success under stress is promising. Extending these approaches to reproductive tissues, particularly those involved in pollen-pistil interactions, fertilization and early seed set, may substantially improve the identification of causal genes and regulatory variants underlying heat-sensitive reproductive traits. This knowledge would provide more precise targets for marker-assisted selection, genomic breeding and genome editing aimed at improving reproductive resilience and yield stability in rice.

Beyond genetic variation, epigenomic regulation provides an additional level controlling reproductive processes under HS. DNA methylation, histone modifications and chromatin accessibility can influence stress-responsive gene expression and may contribute to stress memory in plants. Therefore, integrating methylome profiling, chromatin-level analyses or epigenome-wide association studies (epi-GWAS) with reproductive-stage phenotyping could help identify environmentally responsive regulatory loci associated with heat resilience in female and male tissues. Although the engineering of stress memory in reproductive tissues remains an emerging and largely unexplored strategy, these approaches may provide new targets for improving reproductive thermotolerance under increasingly frequent HS events (Ramakrishnan et al., 2022; Zhou et al., 2025). Recent advances in epibreeding have demonstrated that approaches such as epiGWAs, epigenetic quantitative trait loci (epiQTL) mapping, epigenetic fingerprinting and CRISPR/dCas9-based epigenome editing enable the identification and manipulation of stress-responsive epialleles without altering the DNA sequence, providing new opportunities to improve abiotic stress tolerance (Abdul Aziz and Masmoudi, 2026). Likewise, stress priming has emerged as a promising strategy to induce heritable stress memory, allowing plants to mount faster and stronger responses upon repeated exposure to stress. For example, multigenerational HS in *Arabidopsis thaliana* resulted in stable genetic and epigenetic variants, including differentially methylated positions and regions, associated with enhanced heat tolerance in the progeny (Yadav et al., 2022). Furthermore, primed *O. sativa* seedlings exposed to drought, salinity, or temperature stress displayed altered biochemical and epigenetic profiles that provided enhanced tolerance across generations, indicating heritable stress memory with breeding importance (Kumar et al., 2023). Although evidence specifically linking epigenetic regulation to reproductive thermotolerance remains limited, recent studies in rice provide promising insights. A recent GWA methylome and transcriptome analyses demonstrated that HS during seed development induced 457 differentially methylated regions, primarily located in promoter regions, which were associated with stable changes in gene expression and agronomic traits in the subsequent generation. Differential methylation of genes such as *OsSLB1*, *OsYODA1*, *OsHd1* and *OsAOC1* was maintained in the progeny and correlated with altered tillering, flowering time, floret opening and ultimately a 9.5% increase in grain yield, demonstrating that epigenetic memory can influence reproductive development and agronomic performance across generations (Suriyasak et al., 2026). Complementing these findings, heat-priming experiments in rice revealed that primed plants exposed to HS during flowering exhibited increased spikelet fertility together with extensive proteomic reprogramming involving ribosome biogenesis, protein folding, ROS detoxification and metabolic pathways, providing molecular evidence that stress memory contributes to reproductive thermotolerance (Ju et al., 2025). Furthermore, GWAS analyses of reproductive-stage heat tolerance identified genomic regions, superior haplotypes and candidate genes associated with spikelet fertility and panicle weight under HS, including loci encoding receptor-like kinases, ABA-responsive phosphatases and phosphate transporters, providing valuable genetic resources that could be integrated with epigenomic analyses to identify environmentally responsive regulatory loci controlling reproductive heat resilience (Das et al., 2026).

Together, these studies highlight that combining GWAS with epiGWAS and transcriptomics in reproductive tissues represents a promising strategy for identifying both genetic and epigenetic regulators of heat tolerance. Such multi-layer approaches could facilitate the discovery of environmentally responsive alleles and epialleles underlying stress memory, enabling the development of crops with improved reproductive thermotolerance.

Expanding the scope of research beyond model cultivars such as IR64 and N22 to include landraces, hybrids, and locally adapted varieties will help uncover underexplored genetic diversity and resilience traits (Shi et al., 2022). In parallel, advances in molecular biology, including high-throughput genome sequencing, single-cell transcriptomics, and metabolomics, offer tools to dissect cell-type-specific responses in pistils and ovules (Shi et al., 2022; Visakh et al., 2024). Integrating these approaches into breeding programs, along with new genome editing tools, may strongly accelerate the development of heat-tolerant rice varieties (Chakraborty and Wylie, 2025).

Despite their importance, conventional breeding strategies present inherent limitations, including the unintentional selection of non-target genes and restricted introduction of novel traits due to the inability to cross plants from different species (Tabassum et al., 2021). In the face of the current CC scenario and the increase in global temperatures there is an urgent need for faster and more precise approaches to improve rice reproductive resilience. Genome editing technologies have emerged has powerful tools, enabling targeted genetic modifications with high precision and efficiency (Khalil, 2020). Beyond their potential in crop improvement, precise genome editing tools also enable functional genetic studies under HS conditions.

Among the various gene editing techniques developed, the most promising ones are the CRISPR (Clustered Regularly Interspaced Short Palindromic Repeats) system associated with the Cas9 nickase and other derivative technologies. This method is simple, versatile and highly accurate, enabling the creation of double-strand breaks (DSBs) at specific genomic locations that are repaired by the host’s cellular machinery through either homology-directed repair (HDR) or non-homologous end joining (NHEJ) (Wang et al., 2018b). Several Cas9 variants and alternative systems, such as Cas12a (Cpf1), have expanded the editable genomic space by recognizing non-canonical PAM sequences and improving editing flexibility and efficiency in rice (Endo et al., 2018; Hu et al., 2018; Ren et al., 2019; Alok et al., 2020). These tools have already been successfully applied in rice to modify genes associated with developmental traits and stress responses, demonstrating their feasibility for reproductive-stage-focused studies (Malzahn et al., 2019). These approaches are particularly interesting to fine-tune gene function or regulatory elements associated with HS tolerance, while minimizing unintended genomic alterations. In parallel, RNA-targeting systems such as CRISPR/Cas13 enable transcriptome manipulation without altering the genome, and offer reversible and multiplexed transcript regulation, providing additional opportunities to investigate gene redundancy and dynamic responses during reproductive development, although their broader application still requires careful evaluation of off-target effects (Shuman et al., 2025).

Since reproductive heat tolerance is controlled by multiple interconnected genetic pathways rather than single major genes, multiplex genome editing emerges as a particularly promising strategy for crop improvement. Recent advances in CRISPR technologies allow the simultaneous editing of multiple target genes or regulatory elements, enabling coordinated modification of complex traits that depend on gene networks rather than individual loci (Wang et al., 2018a; Hao et al., 2025). Compared with conventional single-gene editing, multiplex CRISPR approaches provide novel opportunities to simultaneously manipulate pathways controlling multiple genes, thereby addressing the polygenic nature of reproductive thermotolerance. Furthermore, the combination of multiplex genome editing with base editing, prime editing and transcriptional regulation systems (CRISPRa/CRISPRi) enables precise modulation of gene expression without necessarily generating complete loss-of-function alleles, offering greater flexibility for engineering QTLs such as reproductive heat tolerance (Lin et al., 2026). Overall, genome editing technologies provide an essential toolbox to accelerate both functional genomics and breeding efforts aimed at improving rice reproductive resilience to HS. However, their effective implementation depends on the identification of robust candidate genes and regulatory pathways. Integrating GWAS, transcriptomics, epigenomics and precise multiplex genome editing with improved phenotyping and field-relevant stress assays will be critical to translate molecular insights into heat-resilient rice varieties suitable for future climatic conditions (Figure 4).

## Conclusion and future perspectives

HS has a strong impact on rice production, leading to significant losses in both grain quantity and quality. Due to CC and global warming, these problems will become increasingly frequent, leading to a major impact on food security. Understanding the effects of this abiotic stress on plants, particularly during the reproductive phase, is becoming increasingly important, as fruits and seeds are of critical agricultural and economic value.

Despite substantial progress in elucidating plant responses to HS, significant gaps remain in our understanding of rice reproductive responses to HS, from molecular and developmental processes to field-relevant conditions. Particularly, the “blind spot” of rice HS research continues to be the female reproductive organs. To develop new traits, markers, and breeding techniques that will protect reproductive success and yield stability in the face of CC, we must close this knowledge gap.

Future research should integrate precise reproductive phenotyping with GWAS/TWAS, tissue-specific transcriptomics, epigenomics, co-expression networks and functional validation. Applying these multi-layer approaches directly to reproductive tissues will improve the identification of causal genes, regulatory variants and epialleles underlying heat resilience. Combined with multi-environment field trials, locally adapted germplasm and genome editing technologies, this knowledge will support the development of rice varieties capable of maintaining reproductive success and yield stability under increasingly challenging climatic conditions.
